# Hypertension Education Intervention with Ugandan Nurses Working in Hospital Outpatient Clinic: A Pilot Study

**DOI:** 10.1155/2014/710702

**Published:** 2014-12-08

**Authors:** Godfrey Katende, Sara Groves, Kathleen Becker

**Affiliations:** ^1^College of Nursing, Sultan Qaboos University, 123 Muscat, Oman; ^2^School of Nursing, Johns Hopkins University, 525 N. Wolfe Street, Baltimore, MD 21205, USA

## Abstract

Noncommunicable diseases (NCDs) pose a significant global burden in both developed and developing countries. It is estimated that, by 2025, 41.7% of males and 38.7% of females in Sub-Saharan Africa will develop high blood pressure (HBP). This is particularly true in Uganda with hypertensive prevalence rates estimated to range from 22.5% to 30.5%. Coupled with low levels of detection, treatment, and control, hypertension represents a Ugandan public health crisis. An innovative WHO-ISH education program culturally was adapted in a pilot study and focused on knowledge, skills, and attitudes (KSA) of nurses caring for hypertensive patients in an outpatient clinic. Pre-post intervention data was collected and analyzed in which significant improvements were noted on all the three outcome measures. This pilot study demonstrated that nurses' knowledge, skills, and attitudes could be significantly improved with a multimodal education program implemented in a low resource environment.

## 1. Introduction

Noncommunicable diseases (NCDs) pose a significant global burden in both developed and developing countries. Hypertension (HTN) is a significant cardiovascular disorder in Sub-Saharan Africa and is raising epidemic representing a major public health concern. Although hypertension is a growing health concern in Uganda, and like many under resources environments, reliable data on the incidence and its prevalence are lacking [1]. However, it is estimated that, by 2025, 41.7% of males and 38.7% of females in Sub-Saharan Africa will develop high blood pressure (HBP) [2].

In Uganda, a country of 33 million, with an overwhelming morbidity and mortality rate associated with infectious diseases such as HIV/AIDS, tuberculosis, and malaria, is now battling increasing rates of hypertension. The prevalence of hypertension in Uganda has risen dramatically and is estimated to range from 22.5% to 30.5% in the adult population [3, 4]. It is further estimated that nine out of ten people (90%) are unaware of their hypertensive condition [3]. Undetected, poorly managed and uncontrolled hypertension is a major primary and public health care problem contributing to the NCD morbidity and mortality rates in Uganda [3].

High blood pressure is a known contributor to cardiovascular events such as cerebral vascular accidents, myocardial infarctions, congestive heart failure, peripheral vascular disease, and chronic renal failure [5–7]. If untreated or unmanaged, hypertension could lead to significant disability adjusted life years (DALYs), estimated at 8.1% in less developed countries such as Uganda [8, 9]. The disease burden of hypertension is also associated with poor quality of life and loss of productivity, consequently leading to low social economic status. This can potentially affect the social economic status of the whole country [7, 10]. Complicating this health crisis, Uganda is also faced with a woefully shorthanded health work force with estimates of 1.2 physicians and 13.1 nurses and midwives per 10,000 patients [3, 10]. Nurses and midwives make up the largest part of the health workforce in Uganda and play a major role in patient care including screening, taking vital observations, and patient teaching [8]. Moreover, the nurses' educational role has been expanded to make a major contribution to improved patients' lifestyle behaviors, physical activity, weight, stress relief, alcohol intake, medication adherence, and self-efficacy [5, 8, 11–14].

Using the Johns Hopkins Nursing Evidence-Based Practice (JHNEBP) tools for appraising the strength of evidence for both research and nonresearch studies, a comprehensive and rigorous literature review was conducted on hypertension and effective nurse-led interventions [3, 8, 12]. Ten randomized control trials (RCTs), 2 systematic reviews of RCTs without meta-analysis, 3 systematic reviews of RCTs with meta-analysis, 1 descriptive analysis, and 1 nationally recognized expert committee were reviewed. The majority of the systematic reviews (level 1A-B) in the review revealed that nurse-led care for blood pressure control was effective. It should be noted that education alone directed to patients or health professionals was unlikely to influence control of high blood pressure but rather a multifaceted intervention approach was determined to be beneficial [12]. The literature further suggested that determining the correct nursing intervention and appropriate treatment plan to reduce blood pressure for individual patients depended on nurses' basic understanding of the pathophysiology of hypertension, history taking, risk assessment, interpretation of lab results, and the cardiac system [15–17].

The need to duly prepare nurses and physicians working with hypertensive patients has been heavily emphasized to improve care delivery in the developing world [16, 18, 19]. The World Health Organization (WHO) has developed comprehensive hypertension guidelines for low and middle income countries that focus on risk assessment, patient education on diet, weight gain, and other lifestyle changes [7]. These guidelines have not been disseminated nor utilized by Ugandan nurses during the provision of nursing care for hypertensive patients leading to significant variability in nursing practice and missed opportunities for identification and management of HBP. Little is known about studies that address the capacity of the Ugandan nurses in regard to early detection, risk assessment, and patient education on diet, physical activity, and other lifestyle changes for hypertensive patients. This paper draws on quantitative data collected in a nurse-led hypertension pilot project that aimed at enhancing nurses' knowledge, attitude, and skills in early detection, risk assessment, and patient education using culturally adapted nursing hypertension management program based WHO-ISH guidelines and protocols for low and middle income countries.

## 2. Materials and Methods

### 2.1. Design

This was a one group pre-post design using a convenience sample of nurses. The study protocol was approved by the Mulago Hospital Research and Ethics Committee having been determined not to be human subjects' research (MREC: 248). This study was completed from October 2012 to January 2013.

### 2.2. Setting and Sample

Seven (7) nurses from Mulago Hospital's medical outpatient clinic participated in the pilot study. This outpatient clinic is part of Mulago Hospital which is a National Referral and Teaching Hospital with a bed capacity of 1500 beds. Mulago Hospital's medical outpatient clinic is managed by seven (7) nurses, 4 physician assistants, and one consulting physician who attend to an average of 300 patients/day. Of 300 patients, 83% have common infectious diseases such as malaria, respiratory, urinary tract infections, and skin diseases. Averages of 50 patients/day have either diabetes and/or a hypertension diagnosis.

### 2.3. Intervention

The educative interventions were implemented over 3 months and consisted of a hypertension nursing educational program developed and adapted from the WHO-ISH Training Manual for Cardiovascular Risk Assessment and Management (2009) for low and middle income countries. The evidence based interventions focused on improving nurses' knowledge, skills, and attitudes in hypertension management. Knowledge on HBP prevention, detection, risk assessment, patient education, and management was addressed by providing twenty-two (22) hours of didactic classroom sessions. Self-directed learning was bolstered using a CD-ROM on hypertension management previously developed and culturally adapted from the joint Makerere University and Johns Hopkins University team. This CD-ROM was issued to every participating nurse. Participants were also engaged in 1 : 1 clinic sessions that focused on using the WHO risk prediction charts, accurate manual BP measuring techniques, motivation interviewing for patient education on lifestyle changes, and use of the WHO-ISH protocols. English translation to Luganda (local language) was undertaken to ensure all the participating nurses were at the same level of understanding.

### 2.4. Measures

Participant knowledge, skills, and attitudes were measured using pre-post interventions tools. Knowledge was measured using a 10-item multiple choice and multiple response instrument developed by the investigator. The instrument was developed specifically for this study, and asked the nurse participants to respond to questions related to a written case study of a hypertensive Ugandan man with diabetes comorbidity. Before its use, the pre-post knowledge intervention tool was sent to two hypertension experts for review; corrections were incorporated into the final version of the tool and later piloted it with 5 nurses from another nearby hospital. Based on the case study, the questionnaire was composed of questions related to hypertension diagnosis and classification, risk assessment, risk prediction, patient education, and management. In view of being comprehensive, accurate manual blood pressure measuring skills were measured with a 12-step Standardized Blood Pressure Measurement Techniques Skills Checklist [20] retrieved from Tennessee Primary Care Association (TPCA) web page. Nurses' attitudes toward hypertension were measured by using an adapted version of the Respondents' Attitude to Assessment Strategies for Prevention of High Blood Pressure [21]. The Respondents' Attitude to Assessment Strategies for Prevention of High Blood Pressure is a 10-item pre-post attitudes tool which assessed the respondents' attitudes to assessment strategies for prevention of HBP. This tool was initially developed by Dr. Isioma of Nigeria to measure nurses' knowledge and attitudes toward prevention and management of high blood pressure in primary health care centers in Delta state, Nigeria, and was adapted with permission for use in this study. The tool is a 5-point Likert scale type with responses ranging from “strongly agree” to “strongly disagree.” The attitude pre-post tool like the knowledge assessment tool was also sent to the same experts and same nurses before using it with the study participants. The knowledge and attitude pre-post intervention tools all together had 22 items.

### 2.5. Statistical Analysis

Pre-post interventions data on knowledge, skills, and attitudes was entered into the Statistical Package for Social Sciences (SPSS) software version 16. Descriptive statistics were used to describe demographic characteristics. Paired *t*-sample tests were conducted to evaluate the significance of the intervention. Confidence interval was set at 95% and all analyses were two-tailed.

## 3. Results

### 3.1. Demographic Characteristics of Participants

In this pilot study, all the seven nurses working in the outpatient clinic were eligible and invited to participate in the study. Participants were informed of the content on evidence based hypertension management to be completed in 3 months. Participants were also provided with an opportunity to “opt out” in case they were uncomfortable. All seven female nurses from the medical outpatient clinic agreed to participate in the pilot study. Their ages ranged from 37 to 53 years with a mean age of 46.7 years. Approximately 70% received their nursing education in Uganda with at least a diploma level qualification. Mean of years of experience was 2.8 ± 4.1 and less than half (42.9%) had attained a nursing officer (NO) position, the highest nursing position at the outpatient clinic (Table 1).

### 3.2. Knowledge

There was a significant difference in the proportion of nurses who obtained satisfactory answers on the knowledge pre-post interventions test after the educative interventions. Mean test scores prior to the interventions were 62.8% and postinterventions were 82.9%. Paired sample *t*-test indicated a 32% knowledge increase (*P* < 0.009). While the small sample size precluded analysis for each item, it was clear that there was a marked improvement in the nurses' knowledge associated with BP classification, patient education that focused on therapeutic lifestyle changes such as limiting or stopping alcohol intake quitting tobacco use, 30 minutes exercises, losing weight, and eating 400 g of fruits. Similarly, a marked increase in participants' knowledge was observed on risk assessment that included taking blood for cholesterol and serum proteins levels as well as determining the recommended hypertension first line drug used in Uganda. Overall, there was an observed small change in the proportion of nurses who could use of the Risk Prediction Chart at postintervention (Table 2).

### 3.3. Skills

There was a significant difference in the proportion of nurses who correctly performed the required skills as regards accurate manual BP measurement on the Standardized BP Measurement Techniques Skills Checklist. Mean test scores on the accurate BP measurement skills prior to the interventions were 62.7% and after interventions were 94.3%. Paired sample *t*-test indicated a 50% BP skills increase (*P* < 0.000) among participating nurses. Due to small sample size, step by step analysis was not conducted (Tables 3 and 5).

### 3.4. Attitudes

There was a significant difference in the proportion of nurses with positive attitudes on the adapted Respondents' Attitude to Assessment Strategies for Prevention of High Blood Pressure tool. Mean test scores on the attitude test prior to the interventions were 22.4 and after interventions were 39.4. Paired sample *t*-test conducted on the means indicated a 76% increase and improvement in attitudes about assessment strategies used to prevent and treat HBP (*P* < 0.002). Similarly, while the small sample size precluded analysis for each item on the attitude pre-post test, it was clear that there was a marked improvement in nurses' attitudes specifically in regard to patient education, assessment of alcohol and tobacco consumption, importance of documentation of HTN patients' medication, and the need for weight reduction (Tables 4 and 5).

## 4. Discussion

In Uganda, nursing education occurs at the certificate and diploma level with qualifications requiring two to three years of education. Curriculum that focuses on the three competencies, namely, knowledge, attitudes, and skill acquisition, as regards hypertension diagnosis and management occurs during the first year of education. However, the results of the knowledge, skills, and attitudes pretest suggest that nursing curricula should be strengthened. Particularly, this includes evidence based practices to prevent, detect, health-educate, and manage HBP. The concept of evidence based practice (EBP) and translation of best practices is novel to Uganda and has not been incorporated into the nurses' curricula. In this study, as the nurses were introduced to the concept of EBP with the help of the WHO-ISH guidelines and protocols, they became more aware of the available strategies to lower HBP.

The introduction of the hypertension Risk Prediction Charts and risk assessment as an element of EBP were challenging to the participating nurses. Assessing a 10-year risk for cardiovascular diseases using the Risk Prediction Charts is vital and therefore extra effort is required to continue enhancing nurses' knowledge and skills in the use of such evidence based tools. It was evident from the study that the participating nurses' performance on the pre-post interventions test and specifically in regard to risk assessment appeared to be enhanced by use of a multimodal nursing education intervention.

The World Health Organization (WHO) has developed hypertension guidelines for use in low and middle income countries [7]. One of the challenges in changing nursing knowledge and skills is the development of standardized resources. The resources available from WHO are readily available, evidence based, and easily disseminated. When appropriately introduced and culturally adapted, this is an invaluable resource that can assist as standardized protocols for hypertension prevention, risk identification, and risk management in a given population [7]. In this study and prior to the interventions, none of the participating nurses were aware of any existing guidelines or resources be it local, national, or international to facilitate clinical decisions related to hypertension drug treatment. This is because Ugandan nurses lack accessibility and availability to resources related to guidelines and protocols for hypertension management [17]. Access to best care practices is critical to improve and support professionalism of Ugandan nurses and is required for better patients' outcomes.

It was clearly observed in this study that the commitment and enthusiasm of the nurses' ability to learn new approaches to care cannot be overstated. Antidotally, there was improvement in nurses' team cohesion, motivation, and self-efficacy associated with the introduction of a multimodal education approach. Therefore, the concept of team cohesion and team skill building among nurses caring for patients with chronic diseases should further be explored. This may be reflected in the significant improvement (76%) of positive attitudes regarding evidence based interventions for hypertension management. Additional areas to explore are nursing satisfaction with the new professional roles as well as self-efficacy. Lastly, the central role of the patient in terms of satisfaction with care, motivation to engage in therapeutic lifestyle changes, and improved outcomes are important elements to assess in order to provide additional evidence necessary for improved nursing practice. The positive attitude change when used in the enhancement of patient education role forms part of the various evidence based strategies to reduce HBP [17, 22]. It is evident that using a multimodal approach to address various nurse related practice gaps is feasible and could be beneficial when implemented for other chronic diseases with a strategy to regularly update the nurses' knowledge, skills, and attitudes as regards translation of best evidence into their practice [18] for better patients' outcomes [22].

## 5. Implications for Nursing Practice

Findings from this pilot study highlight the importance of using an evidence based guideline to improve nurses' knowledge, skills, and attitudes in blood pressure management. Risk assessment knowledge and skills must be emphasized during any educational intervention and organized to accentuate short, medium, and long term benefits of HBP detection, prevention, and management. Evidence based strategies that improve nurses' capacity to manage HBP in a low resource primary setting are also imperative. Rolling out of the study intervention could improve nurses' practices especially those working in outpatient care settings.

## 6. Recommendation for Future Nursing Research

Self-efficacy and barriers to gaining knowledge, skills, and attitudes among nurses need to be explored. Future research could focus on other groups of nurses and not necessarily working in outpatient clinics to overcome sample size issues and compare hypertension knowledge, skills, and attitudes in other areas of specialization. Similarly, there is need to identify and select valid and reliable instruments for use in assessing outcome measures.

## 7. Conclusion 

After 3 months of implementing the nurses' hypertension management educative interventions program, knowledge, skills, and attitudes regarding prevention, detection, risk assessment, patient education, and HBP management increased significantly. The pilot study demonstrated the feasibility of implementing a multimodal evidence-based educational intervention in a low resource environment.

## Figures and Tables

**Table 1:** Demographic characteristics.

Variable	*N* = 7
Age, mean (SD)	46.7 (5.9)
Sex, % females	100%
Years employed, mean (SD)	2.8 (4.1)
Level of education, %	
Certificate	28.6%
Diploma	71.4%
Highest position attained, %	
Assistant in charge	14.3%
Enrolled nurse	14.3%
In charge domiciliary	14.3%
Nursing officer (NO)	42.9%
NO^*^ in charge	14.3%

NO is a registered nurse at diploma level.

NO^*^ is a double trained registered nurse with an administrative role similar to charge nurse.

SD: standard deviation.

**Table 2:** Proportion of participants' correct responses on knowledge pre-post test.

Variable A—knowledge *N* = 7	Percent (pre)	Percent (post)
(1) Blood pressure (BP) classification	85.7	100
(2) Life style changes		
(i) Stop all alcohol intake	57.1	100
(ii) Quit tobacco use	14.3	100
(iii) Take baby aspirin	85.7	85.7
(iv) Losing weight	71.4	85.7
(v) Eat 400 g of fruits and vegetables	0	57.1
(3) Risk assessment		
(i) Taking single BP reading	14.3	42.9
(ii) Taking blood cholesterol levels	57.1	85.7
(iii) Taking blood for serum protein levels	28.6	100
(4) Using risk prediction chart	0	14.3
(5) Recommended hypertension first line drug in Uganda	0	100
(6) Weight reduction is rather tasking to be an effective strategy	71.4	14.3

Data extracted from primary source.

**Table 3:** Proportion of participants' correct responses on accurate BP measuring skills with the help of a Standardized Blood Pressure Measurement Techniques Skills Checklist.

Variable—BP measure skills	Percent (pre)	Percent (post)
(1) Asks patient about eating, drinking caffeine, smoking	0	71.4
(2) Locates brachial artery	85.7	100
(3) Rechecks blood pressure		
(i) Waits one to two min before recheck	28.6	85.7
(ii) Waits for 15 seconds after deflation	28.6	100
(4) Positions patient's bare arm on a hard surface	42.9	71.4
(5) Obtains systolic (phase 1) and diastolic BP values (phase 5, or 4)	28.6	100
(6) Inflates cuff at 10 mmhg increments until the patient's pulse disappears	42.9	71.4

Data extracted from primary source.

**Table 4:** Proportion of participants' perceived responses on the attitudes pre-post test using Respondents' Attitude to Assessment Strategies for Prevention of High Blood Pressure tool.

Variable *N* = 7	Percent (pre)	Percent (Post)
(1) Patient education intervention to reduce BP	85.7	100
(2) Disagreed that alcohol and tobacco consumption status is personal and should not be discussed with patient	85.7	42.9
(3) Regular documentation of HT patient medication a waste of time	71.4	14.3
(4) Weight reduction is rather tasking to be an effective strategy	71.4	14.3
(5) Overall attitude change from baseline	**76**%	

Data extracted from primary source.

**Table 5:** Comparison of outcome measures before and after educative intervention.

Outcome variable (*N* = 7)	Preintervention	Postintervention		% change
Knowledge %, mean (SD)	62.8% (13.8)	82.9% (3.6)	*P* < 0.009^*^	32%
Attitude, mean (SD)	22.4 (6.0)	39.4 (4.7)	*P* < 0.002^*^	76%
Skills %, mean (SD)	62.7 (9.2)	94.3% (7.5)	*P* < 0.000^**^	50%

Data extracted from the primary source.

SD: standard deviation; *N*: total number of participants; CI 95%: confidence interval, ^*^ *P* < 0.05, ^**^ *P* < 0.001, two-tailed.

## Limitations

The outpatient clinic only has a small number of nurses for whom all participated in the pilot study and therefore may limit the interpreting of the findings. This pilot study should be implemented in various outpatient clinics to achieve larger samples that could be used for comparison of patient outcomes and also test the reliability of the tools used.

## References

[1] Twagirumukiza M., De Bacquer D., Kips J. G., de Backer G., Stichele R. V., van Bortel L. M. (2011). Current and projected prevalence of arterial hypertension in sub-Saharan Africa by sex, age and habitat: an estimate from population studies. *Journal of Hypertension*.

[2] Addo J., Smeeth L., Leon D. A. (2007). Hypertension in sub-Saharan Africa: a systematic review. *Journal of Hypertension*.

[3] Maher D., Waswa L., Baisley K., Karabarinde A., Unwin N. (2011). Epidemiology of hypertension in low-income countries: a cross-sectional population-based survey in rural Uganda. *Journal of Hypertension*.

[4] Wamala J. F., Karyabakabo Z., Ndungutse D., Guwatudde D. (2009). Prevalence factors associated with hypertension in Rukungiri District, Uganda—a community-based study. *African Health Sciences*.

[5] Bakris G., Hill M., Mancia G., Steyn K., Black H. R., Pickering T., De Geest S., Ruilope L., Giles T. D., Morgan T., Kjeldsen S., Schiffrin E. L., Coenen A., Mulrow P., Loh A., Mensah G. (2008). Achieving blood pressure goals globally: five core actions for health-care professionals. A worldwide call to action. *Journal of Human Hypertension*.

[6] Kearney P. M., Whelton M., Reynolds K., Muntner P., Whelton P. K., He J. (2005). Global burden of hypertension: analysis of worldwide data. *The Lancet*.

[7] WHO (2010). *Package of Essential Noncommunicable (PEN) Disease Interventions for Primary Health Care in Low-Resource Settings*.

[8] Glynn L. G., Murphy A. W., Smith S. M., Schroeder K., Fahey T. (2010). Interventions used to improve control of blood pressure in patients with hypertension. *The Cochrane Database of Systematic Reviews*.

[9] Fulwood R., Guyton-Krishnan J., Wallace M., Sommer E. (2006). Role of community programs in controlling blood pressure. *Current Hypertension Reports*.

[10] WHO (2011). *WHO-NCD Country Profiles, Uganda*.

[11] Hacihasanoğlu R., Gözüm S. (2011). The effect of patient education and home monitoring on medication compliance, hypertension management, healthy lifestyle behaviours and BMI in a primary health care setting. *Journal of Clinical Nursing*.

[12] Fahey T., Schroeder K., Ebrahim S. (2005). Educational and organisational interventions used to improve the management of hypertension in primary care: a systematic review. *British Journal of General Practice*.

[13] Clark C. E., Smith L. F., Taylor R. S., Campbell J. L. (2011). Nurse led interventions to improve control of blood pressure in people with hypertension: systematic review and meta-analysis. *British Medical Journal*.

[14] Bengtson A., Drevenhorn E. (2003). The nurse's role and skills in hypertension care: a review. *Clinical Nurse Specialist*.

[15] Chummun H. (2009). Understanding changes in cardiovascular pathophysiology. *British Journal of Nursing*.

[16] Abolfotouh M. A., Soliman L. A., Abolfotouh S. M., Raafat M. (2011). Knowledge and practice of PHC physicians toward the detection and management of hypertension and other CVD risk factors in Egypt. *International Journal of Hypertension*.

[17] Chen H. L., Liu P. F., Liu P. W., Tsai P. S. (2011). Awareness of hypertension guidelines in Taiwanese nurses. A questionnaire survey. *Journal of Cardiovascular Nursing*.

[18] Silava S. S. B. E., Colosimo F. C., Pierin A. M. G. (2010). The effect of educational interventions on nursing team knowledge about arterial hypertension. *Revista da Escola de Enfermagem da USP*.

[19] Kaufman N. D., Rajataramya B., Tanomsingh S., Ronis D. L., Potempa K. (2012). Nurse preparedness for the non-communicable disease escalation in Thailand: a cross-sectional survey of nurses. *Nursing and Health Sciences*.

[20] Tennesse Primary Care Association (TPCA) Standardized Blood Pressure Measurement Techniques Skills Checklist. http://www.tnpcaeducation.org/resourcelibrary/clinical/Hypertension/Skills%20Checklist.pdf.

[21] Isoma M. O. (2012). Nurses knowledge and attitude in the prevention and management of high blood pressure in primary health care centers in Delta state, Nigeria. *Global Advanced Research Journals of Nursing and Midwifery*.

[22] Stewart J., Dyas J., Brown K., Kendrick D. (2006). Achieving blood pressure targets: lessons from a study with practice nurses. *Journal of Diabetes Nursing*.

